# High *Streptococcus pneumoniae* colonization prevalence among HIV-infected Kenyan parents in the year before pneumococcal conjugate vaccine introduction

**DOI:** 10.1186/s12879-015-1312-2

**Published:** 2016-01-16

**Authors:** Laura M. Conklin, Godfrey Bigogo, Geofrey Jagero, Lee Hampton, Muthoni Junghae, Maria da Gloria Carvalho, Fabiana Pimenta, Bernard Beall, Thomas Taylor, Brian Plikaytis, Kayla F. Laserson, John Vulule, Chris Van Beneden, Cynthia G. Whitney, Robert F. Breiman, Daniel R. Feikin

**Affiliations:** 1 Division of Bacterial Diseases, Centers for Disease Control and Prevention, Atlanta, GA USA; 2 Kenya Medical Research Institute/Centers for Disease Control and Prevention, Kisumu, Kenya; 3 Centre for Global Health Research, Kenya Medical Research Institute, Kisumu, Kenya; 4 Respiratory Diseases Branch, Centers for Disease Control and Prevention, 1600 Clifton Road NE, MS C-25, Atlanta, GA 30333 USA; 5 KEMRI/CDC Research Collaboration, P. O. Box 1578, Kisumu, 40100 Kenya

**Keywords:** *Streptococcus pneumoniae*, Pneumococcus, Nasopharyngeal colonization, HIV, Kenya, Africa, PCV

## Abstract

**Background:**

*Streptococcus pneumoniae* is a leading cause of pneumonia, meningitis and sepsis in developing countries, particularly among children and HIV-infected persons. Pneumococcal oropharyngeal (OP) or nasopharyngeal (NP) colonization is a precursor to development of invasive disease. New conjugate vaccines hold promise for reducing colonization and disease.

**Methods:**

Prior to introduction of 10-valent pneumococcal conjugate vaccine (PCV10), we conducted a cross-sectional survey among HIV-infected parents of children <5 years old in rural Kenya. Other parents living with an HIV-infected adult were also enrolled. After broth enrichment, NP and OP swabs were cultured for pneumococcus. Serotypes were identified by Quellung. Antimicrobial susceptibility was performed using broth microdilution.

**Results:**

We enrolled 973 parents; 549 (56.4 %) were HIV-infected, 153 (15.7 %) were HIV-uninfected and 271 (27.9 %) had unknown HIV status. Among HIV-infected parents, the median age was 32 years (range 15-74) and 374/549 (68 %) were mothers. Pneumococci were isolated from 237/549 (43.2 %) HIV-infected parents and 41/153 (26.8 %) HIV-non-infected parents (*p* = 0.0003). Colonization with PCV10 serotypes was not significantly more frequent in HIV-infected (12.9 %) than HIV-uninfected parents (11.8 %; *p* = 0.70). Among HIV-infected parents, cooking site separate from sleeping area and CD4 count >250 were protective (OR = 0.6; 95 % CI 0.4, 0.9 and OR = 0.5; 95 % CI 0.2, 0.9, respectively); other associations were not identified. Among 309 isolates tested from all parents, 255 (80.4 %) were penicillin non-susceptible (MIC ≥0.12 μg/ml).

**Conclusions:**

Prevalence of pneumococcal colonization is high among HIV-infected parents in rural Kenya. If young children are the pneumococcal reservoir for this population, PCV10 introduction may reduce vaccine-type colonization and disease among HIV-infected parents through indirect protection.

## Background

*Streptococcus pneumoniae* (pneumococcus) is a leading cause of pneumonia, meningitis and sepsis among children and adults in developing countries [1, 2]. Pneumococci colonize the upper respiratory tract, which is a precursor state to pneumonia and invasive pneumococcal disease (IPD). Transmission is common from person-to-person through respiratory secretions, particularly within families and other groups in which people are in close contact [3–5]. Groups at highest risk for IPD after pneumococcal acquisition include young children, the elderly and immuno-compromised individuals such as those infected with Human Immunodeficiency Virus (HIV). Persons infected with HIV are approximately 25 to 50-fold more likely to develop IPD than are HIV-uninfected persons [6–8].

While contact with young children is a well-established risk factor for both pneumococcal colonization and disease among all adults [9, 10], it is a particularly important determinant of pneumococcal colonization and disease among HIV-infected adults [11]. HIV-infected adults are more likely to have disease caused by serotypes associated with invasive disease in children (e.g. 6B, 9 V, 14, 19 F, 23 F) than HIV-uninfected adults [12], and HIV-infected mothers are more likely to have disease caused by pediatric serotypes than HIV-infected fathers [13, 14].

The introduction of the 7-valent pneumococcal conjugate vaccine (PCV7) among U.S. children in 2000 led to a 94 % decrease in vaccine-type IPD among all children <5 years from 1998–2003 [15]. It also led to decreases in IPD among adults, including a 25 % decline in all-serotype IPD incidence among HIV-infected adults living with AIDS [7, 10]. This indirect or “herd” effect in unvaccinated persons occurred because vaccinated children were less likely to be colonized with PCV7 serotypes and were therefore less likely to transmit them to unvaccinated persons [16, 17].

It is unknown whether the substantial indirect vaccine effect seen among high-risk groups in the U.S. will also be seen in African countries with high HIV seroprevalence. In January 2011, Kenya became the third African country to introduce pneumococcal conjugate vaccine into its national immunization program, and the first to utilize the 10-valent formulation (PCV10) which expanded coverage to include PCV7 serotypes (4, 6B, 9 V, 14, 18C, 19 F, 23 F) plus serotypes 1, 5, and 7 F. To better understand the changes in pneumococcal ecology among high risk groups before and after introduction of PCV10, we designed a study of pneumococcal colonization among HIV-infected parents of young children in a high HIV prevalence area of western Kenya. In this report, we describe the baseline prevalence of pneumococcal colonization, including serotype distribution, antibiotic resistance, and risk factors for colonization.

## Methods

### Study setting

This study utilized two ongoing surveillance systems in Asembo, western Kenya, to select and enroll participants—the western Kenya Health and Demographic Surveillance System (HDSS) and Population-Based Infectious Disease Surveillance (PBIDS) program. Both were established through collaboration between the Kenya Medical Research Institute (KEMRI) and Centers for Disease Control and Prevention (CDC) [18, 19]. The Asembo area is mostly poor and is located in a rural province with one of the highest HIV prevalence rates in Kenya. HDSS collects demographic data on the population, including health status, socioeconomic status, and education. In 2007, 14.9 % of adults ages 15–64 years in Asembo were HIV-infected [20]. Since 2005, residents of 33 HDSS villages within the Asembo HDSS area have also been enrolled in PBIDS, which measures morbidity in the community and at the hospital [18]. Home-based counseling and testing (HBCT) for HIV occurred throughout the Asembo area in 2008–2009; these data were linkable to HDSS data and used to identify HIV-infected persons for this study [20]. HIV status was determined using two parallel HIV rapid tests, as previously described [20]. Participants provided written consent for any testing performed, and for linkage of HIV results to their HDSS and PBIDS records.

### Cross-sectional survey

We performed a cross-sectional survey among HDSS residents who were parents of a child <5 years of age and resided within the 33 PBIDS villages and 13 additional adjacent HDSS villages in the Asembo area. We used HDSS records to identify living compounds where at least one HIV-infected parent of a child under 5 years of age resided. To maintain confidentiality on HIV testing status, HDSS village reporters approached the selected compound and invited all parents of children under 5 years of age (regardless of HIV status) residing there to participate in the study. Interested persons were referred to St. Elizabeth’s Mission Hospital clinic for enrollment. Upon enrollment, data on household characteristics, recent respiratory illness, smoke exposure, cooking practices, and antibiotic usage were collected. Additional demographic data and HIV-indicators (HIV status, CD4 counts, use of highly active antiretroviral therapy [HAART], and attendance at an HIV clinic) were obtained through HDSS and PBIDS databases. For these data, we attempted to obtain the most recent information reported prior to sample collection. The survey was conducted during October 29–December 23, 2009.

### Laboratory methods and definitions

Polyester-tipped swabs were swept over the posterior oropharynx (OP) and tonsils, and calcium alginate swabs were inserted into the posterior nasopharynx (NP) and rotated 360°, as previously described [21]. Both NP and OP swabs were collected from each participant. Swabs were immediately placed in separate vials containing skim milk-tryptone-glucose-glycerol (STGG) transport medium and placed in a cool box as per World Health Organization consensus methods [22]. Within 8 h, specimens were vortexed and placed in a liquid nitrogen container. The next morning specimens were transported approximately 50 km to the KEMRI/CDC laboratory and stored at −70 °C.

Pneumococcal isolation was conducted at the KEMRI-CDC laboratory in Kisumu, Kenya by adding 200 μl of NP-STGG 200 μl of OP-STGG from each individual in an enrichment broth step following methods previously described [23]. Any pneumococcal alpha-hemolytic colony potentially identifiable as *S. pneumoniae* was subjected to optochin susceptibility and bile solubility testing [24]. In cases where more than one potential pneumococcal colony type was identified per plate, representatives of each colony type were subjected to testing. Pneumococcal isolates were then transported on dry ice to the CDC laboratory in Atlanta, GA for serotyping. Serotypes for the pneumococcal isolates were obtained by latex agglutination and Quellung reaction testing. Antimicrobial susceptibility testing for commonly used antibiotics was performed at KEMRI-CDC or CDC-Atlanta laboratories by broth microdilution (Trek Diagnostics, Cleveland OH) according to the manufacturer’s instructions. Susceptibility was determined using Clinical and Laboratory Standards Institute (CLSI) criteria for minimum inhibitory concentration (MIC) from 2012 for non-beta lactams and 2007 for penicillin (≥0.12 μg/ml), which we felt was most biologically relevant for carriage where reduced susceptibility may provide a selective advantage. Intermediate and resistant isolates were designated as “non-susceptible”.

We categorized pneumococcal colonization by serotypes present in either PCV10 (serotypes 1, 4, 5, 6B, 7 F, 9 V, 14, 18C, 19 F, 23 F) or the 13-valent PCV (PCV10 serotypes plus serotypes 3, 6A and 19A) vaccine formulations. When multiple pneumococcal serotypes were identified from a specimen, participants were classified as colonized by a vaccine-serotype if at least one serotype was included in the vaccine. The isolate with the highest MIC was used in reporting of antimicrobial resistance when more than one isolate was detected.

### Data management and analysis

Analyses were performed using SAS software (version 9.3; SAS institute). We categorized participants by HIV-status (HIV-infected, HIV-uninfected, or HIV-unknown) and used data on CD4 counts, history of use of HAART, and last HIV-clinic attendance, when available. We defined an ‘isolate’ as a pneumococcal strain of a particular serotype from a participant. For example, if two colonies were selected from a plate and had the same serotype, this was considered 1 isolate; colonies of 2 different serotypes were considered 2 isolates. We calculated a serotype diversity index (SDI) by dividing the number of serotypes detected by the total number of isolates, such that the maximum diversity score would be 1.00 and least would be approaching 0. We performed univariable and multivariable logistic regression to assess the association of various risk factors with colonization among participants, accounting for compound as a repeated measure. Interactions with antibiotic usage, age, and gender were explored. Odds ratios with 95 % confidence intervals were calculated.

### Ethical considerations

This study was approved by both KEMRI and CDC ethical committees. All participants gave written informed consent.

## Results

We identified 772 HIV-infected parents of children <5 years old living among 436 compounds in the HDSS database. Of these, 549 (71.0 %) were enrolled in the study. The primary reason for non-enrollment in the study was out-migration from the HDSS area. Persons enrolled did not differ significantly from those not enrolled by gender, although non-responders were slightly younger (median age 28 years, compared to 32 years among responders; *p* < 0.0001). An additional 424 parents (153 HIV-uninfected and 271 of unknown HIV status) living with an HIV-infected parent also participated in the study.

The age range among enrolled HIV-infected parents was 15–74 years, and 68 % were mothers (Table 1). The median number of children <5 years old living in each household was 1 (range 1–4). A total of 141 (25.7 %) reported a current cough, and 74 (13.5 %) reported a fever within the previous 24 h. A large proportion reported antimicrobial usage on the day of swabbing (*n* = 169, 30.8 %), the majority of which included cotrimoxazole (161 of 169, 95.2 %). Among 200 parents for whom details on HIV indicators were available, 106 (53.8 %) were enrolled at an HIV care and treatment clinic, 23 (11.6 %) were on HAART, and 44 (22.0 %) had CD4 counts <250.

**Table 1:** Characteristics of HIV-infected parents of children <5 years old in a survey of *Streptococcus pneumoniae* colonization in Western Kenya (*n* = 549)^a^

Characteristic	N (%)
Female gender	374 (68.1)
Age, median years (range)	32 (17–74)
< 30 years	188 (34.3)
30–39 years	269 (48.9)
> =40 years	92 (16.8)
Wealth quintile^b^	
1 poorest	32 (11.3)
2	50 (17.6)
3	69 (24.3)
4	57 (20.1)
5 least poor	76 (26.8)
Currently employed	112 (20.6)
Number of years living in Asembo, median (range)	14 (0–73)
Number of compounds	460
Number of people in compound, median (range)	8 (1–38)
Median number of rooms used for sleeping	1 (1–6)
Number of children under 5 in the home, median (range)	1 (1–4)
1	328 (59.7)
2	185 (33.7)
≥ 3	36 (6.6)
Number of children in home attending school, median (range)	2 (0–13)
none	67 (12.2)
≥ 1	482 (87.8)
Recent illness	
Current cough	141 (25.7)
Cough within 30 days	171 (31.3)
Pneumonia within 30 days	16 (2.9)
Fever within 24 hours	74 (13.5)
Fever within 30 days	171 (31.2)
Smoke exposure	
Smokes tobacco	29 (5.3)
Tobacco smoke in the home	99 (18.2)
Type of fuels used for cooking	
Firewood	540 (98.4)
Charcoal	207 (37.7)
Other^c^	23 (4.2)
Area used for cooking	
Same area used for sleeping	243 (44.3)
Dedicated separate building	235 (42.8)
Outside the house	45 (8.2)
Within the house, separate room	26 (4.7)
Antibiotic use^d^	
Use of any antibiotic within 7 days	313 (57.0)
Current use of any antibiotic	169 (30.8)
Current use of cotrimoxazole	161 (29.3)
Attends HIV clinic^e^	106 (53.8)
HAART use^e^	23 (11.6)
CD4 count, median (range)^e, f^	445 (21–1378)
< 250	44 (21.9)
250–499	71 (35.3)
> 500	86 (42.8)

^a^Percentages reflect missing data

^b^Wealth indices categorized into five quintiles: 1 (Poorest) to 5 (Least Poor)

^c^Includes gas, kerosene, paraffin, and dung

^d^Current use refers to the day of interview

^e^Data available for 201 respondents

^f^Number of days since most recent CD4 count ranged from 166 before date of interview to 840 days after (median 394 days before date of interview)

Pneumococcal colonization was 38.6 % among all participants (Table 2), although prevalence was significantly higher among HIV-infected parents (43.2 %) compared to HIV-uninfected parents (26.8 %) living in the same compounds (*p* = 0.0003). Colonization with PCV10 serotypes was not significantly more frequent among HIV-infected (12.9 %) than HIV-uninfected parents (11.8 %; *p* = 0.70). Among pneumococcal colonizers, the proportion of isolates that were PCV10 serotypes was lower among HIV-infected parents (30.0 %) than among HIV-uninfected parents (43.9 %), but the difference was not statistically significant (*p* = 0.08).

**Table 2:** Pneumococcal colonization among parents of children <5 years of age living in the same compound in Western Kenya, by HIV-status (N = 973)

	HIV- Infected only	HIV-uninfected only	HIV-unknown only	All Participants
	N (%)	N (%)	N (%)	N (%)
Number of participants	549	153	271	973
Pneumococcal colonization, any type^a^	237 (43.2)	41 (26.8)	97 (35.8)	375 (38.5)
PCV10 type only	71 (12.9)	18 (11.8)	26 (9.6)	115 (11.8)
PCV13-type only	108 (19.7)	24 (15.7)	44 (16.2)	176 (18.1)
Total number of isolates detected^b^	242	41	98	381
Number of different pneumococcal serotypes detected per person^c,a^				
0	312 (56.8)	112 (73.2)	174 (64.2)	598 (61.4)
1	232 (42.3)	41 (26.8)	96 (35.4)	369 (38.0)
2	5 (0.9)	0	1 (0.4)	6 (0.6)
Total number of different pneumococcal serotypes detected^e^	35	16	30	41
Serotype diversity index^d,e^	0.14	0.39	0.31	0.11

^a^ *p*-value comparing HIV-infected compared to HIV-uninfected <0.001

^b^Any pneumococcal colony suspected of being alpha-hemolytic was selected for identification by susceptibility to optochin and bile solubility. In some cases, more than one colony was identified per plate

^c^No participant had >2 serotypes identified

^d^Serotype diversity index = total number of different pneumococcal serotypes detected divided by total number of isolates detected; Maximum diversity is 1.0 and least diversity is 0

^e^Does not include non-typeable isolates: HIV-infected (1), HIV-uninfected (2), HIV-unknown (3)

**Fig. 1:**
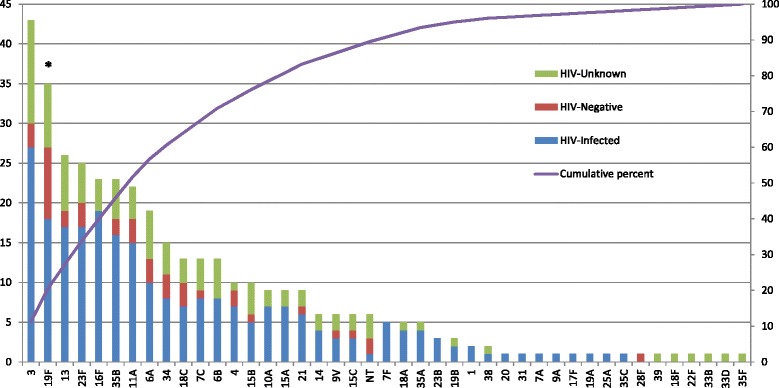
Serotypes associated with pneumococcal colonization among parents of children under 5 years old living in the same compound in rural Kenya, by HIV status (n = 381). *p-value comparing HIV-infected compared to HIV-uninfected <0.01 for serotype 19 F

We detected 35 different serotypes from 242 isolates among HIV-infected parents; five (0.9 %) individuals carried 2 serotypes each. Although fewer numbers of serotypes were detected among HIV-uninfected parents (*n* = 16), the serotype diversity index (SDI) was higher overall when compared to HIV-infected parents (0.39 compared to 0.14) (Table 2). Serotypes 3 (11.2 %), 16 F (7.9 %), and 19 F (7.4 %) were the most common isolates detected among HIV-infected parents (Fig. 1). No significant differences in colonization with specific serotypes was observed with the exception of serotype 19 F, which was observed less frequently among HIV-infected parents than HIV-uninfected parents (7.4 % vs 22.0 %; *p* = 0.003).

On multivariable analysis, high CD4 count was associated with significant decrease in colonization among HIV-infected persons (Table 3). HIV-infected parents with CD4 count ≥250 were less likely to be colonized than those with lower CD4 counts (OR = 0.5; 95 % CI 0.2, 0.9). We also observed a protective effect of cooking location being separate from sleeping location (OR = 0.6; 95 % CI 0.4, 0.9). We did not observe a significant association between colonization and smoking tobacco, the number of children <5 years old in the home, the number of children attending school, the number of people living in the compound, wealth quintile, type of cooking fuel used, or self-reported fever within 24 h of sample collection. HIV infected parents were 2.1 times more likely to be colonized than HIV-uninfected parents (95 % CI 1.44, 3.03; data not shown), controlling for other factors. We did not see any other differences in colonization risk factors by HIV status. Interactions with gender or amoxicillin use (current, within 7 days, or within 30 days of sample collection), gender were also not observed. Although age less than 40 years was protective in univariable analyses (OR = 0.6; 95 % CI 0.4, 0.9), this did not hold true in multivariable models.

**Table 3:** Factors associated with pneumococcal colonization among HIV-infected parents of children <5 years old Kenya (N = 549)

	Colonized	Not Colonized	OR (95 % CI)^a^
	N = 237	N = 311	
	N (%)	N (%)	
Recent Illness			
No fever within 24 hours	199 (84.0)	274 (88.4)	*referent*
Self-reported fever within 24 hours	38 (16.0)	36 (11.6)	1.5 (0.9, 2.6)
Number of children <5 years old in the home			
1	145 (61.2)	182 (58.5)	*referent*
2	77 (32.5)	108 (34.7)	0.9 (0.6,1.3)
> 2	15 (6.3)	21 (6.8)	0.9 (0.5, 1.9)
Number of children in school			
0	24 (10.1)	43 (13.8)	*referent*
≥ 1	213 (89.9)	268 (86.2)	1.6 (0.9, 2.6)
Cooking location			
Same as sleeping area	122 (51.5)	120 (38.6)	*referent*
Other location separate from sleeping area	115 (48.5)	191 (61.4)	0.6 (0.4, 0.9)
Tobacco smoke exposure^b^			
No smoker in the household	190 (81.2)	255 (82.2)	*referent*
Smoker in the household	44 (18.8)	55 (17.8)	1.1 (0.7, 1.6)
Multiple Correspondence Analysis (MCA) quintile^b, c^			
< 3	40 (31.5)	42 (26.7)	*referent*
≥ 3	87 (68.5)	115 (73.3)	0.8 (0.5, 1.4)
CD4 Count^b^			
CD4 < 250	25 (28.7)	19 (16.8)	*referent*
CD4 ≥ 250	62 (71.3)	94 (83.2)	0.5 (0.2, 0.9)
HAART^b^			
No or unknown HAART use	76 (88.4)	100 (88.5)	*referent*
HAART use	10 (11.6)	13 (11.5)	1.1 (0.5, 2.4)

^a^Adjusted for compound as a repeated measure, amoxicillin use within 7 days, age, and gender

^b^Percentages reflect missing data

^c^Wealth indices categorized into five quintiles: 1 (Poorest) to 5 (Least Poor)

Among 309 isolates tested for antimicrobial resistance among all parents enrolled, a large proportion were non-susceptible to one or more antibiotics: 308 (99.0 %) to cotrimoxazole, 255 (80.4 %) to penicillin, and 78 (24.7 %) to tetracycline (Tables 4 and 5). For other antibiotics (levofloxacin, ceftriaxone, chloramphenicol, erythromycin, clindamycin), the proportion non-susceptible was 0–1.3 %. Antimicrobial susceptibility patterns were similar across parent groups; although a greater proportion of the isolates carried by HIV-infected parents were non-susceptible to penicillin (83.4 %) when compared to isolates from HIV-negative parents (67.7 %), this difference was not statistically significant (*p* = 0.09; data not shown). Most of the penicillin non-susceptible isolates were intermediately resistant (MICs 0.12–1.0 μg/ml) rather than fully resistant (≥2 μg/ml) to penicillin.

**Table 4:** Antimicrobial susceptibility of 309 pneumococcal isolates obtained from nasopharyngeal and oropharyngeal swabs collected from 375 parents of children under 5 years of age in Western Kenya, by antiobiotic.

Antibiotic	Susceptible		Intermediate		Resistant	
	*Break-point* ^*a*^	N (%)	*Break-point*	N (%)	*Break-point*	N (%)
Penicillin	*≤0.06*	60 (19.4)	*0.12-1*	249 (78.6)	*≥2*	6 (1.9)
Chloramphenicol	*≤4*	305 (98.7)	*n/a*	n/a	*≥8*	4 (1.3)
Levofloxacin	*≤2*	312 (100)	*4*	0	*≥8*	0
Erythromycin	*≤0.25*	305 (98.7)	*0.5*	1 (0.3)	*≥1*	3 (1.0)
Ceftriaxone	*≤1*	309 (100)	*2*	0	*≥4*	0
Tetracycline	*≤2*	232 (75.3)	*4*	12 (3.9)	*≥8*	64 (20.8)
Cotrimoxazole	*≤0.5/9.5*	3 (1.0)	*1/19–2/38*	13 (4.2)	*≥4/76*	291 (94.8)
Clindamycin	*≤0.25*	308 (99.7)	*0.5*	0	*≥1*	1(0.3)

^a^Breakpoints defined using Clinical and Laboratory Standards Institute (CLSI) guidelines 2007 for penicillin and 2012 for all other antibiotics

*n/a* not applicable

**Table 5:** Number of non-susceptible (i.e. intermediate or resistant) isolates detected among 309 pneumococcal isolates from parents of children under 5 years of age in Western Kenya, by serotype included in the PCV10 or PCV13 vaccine* [N (%)]

	Serotype													
	1	3	4	6A	6B	7 F	9 V	14	18C	19A	19 F	23 F	Any PCV10	Any PCV13
Number of isolates	2	39	7	16	8	4	6	4	10	1	32	25	98	154
Antibiotic														
Penicillin	1 (50)	38 (97)	3 (43)	9 (56)	8 (100)	4 (100)	4 (66)	4 (100)	3 (30)	1 (100)	31 (97)	20 (80)	78 (80)	125 (81)
Chloramphenicol	0	0	0	0	0	0	2 (33)	0	0	0	0	1 (4)	3 (3)	3 (2)
Levofloxacin	0	0	0	0	0	0	0	0	0	0	0	0	0	0
Erythromycin	0	1 (3)	0	0	0	0	0	0	0	0	1 (3)	0	1 (1)	2 (1)
Ceftriaxone	0	0	0	0	0	0	0	0	0	0	0	0	0	0
Tetracycline	0	26 (66)	1 (14)	0	1 (13)	0	2 (33)	4 (100)	0	0	4 (13)	1 (4)	13 (13)	39 (25)
Cotrimoxazole	2 (100)	37 (97)	7 (100)	16 (100)	8 (100)	4 (100)	6 (100)	4 (100)	8 (80)	1 (100)	25 (78)	25 (100)	89 (91)	143 (93)
Clindamycin	0	1 (3)	0	0	0	0	0	0	0	0	0	0	0	1 (1)

*PCV10* Ten-valent pneumococcal conjugate vaccine (serotypes 1, 4, 5, 6B, 7 F, 9 V, 14, 18C, 19 F, 23 F), *PCV13* Thirteen-valent pneumococcal conjugate vaccine (serotypes 1, 3, 4, 5, 6A, 6B, 7 F, 9 V, 14, 18C, 19A, 19 F, 23 F)

^a^No serotype 5 isolates were detected

## Discussion

PCV introduction in Africa has accelerated in recent years, yet few published data document the baseline prevalence of pneumococcal disease or colonization among groups not targeted to receive vaccine, particularly adults with HIV infection. The 43.2 % pneumococcal colonization rate that we observed among HIV-infected adults is similar to a previous study conducted in Kenya among HIV clinic attendees (34.6 %) [25], but higher than in studies conducted among HIV clinic attendees in Uganda (18.0 %), mineworkers in South Africa (8.8 %), and mothers of young infants in both South Africa (20.2 %) and Zambia (11.4 %) [26–29]. The overall colonization rate of 38.6 % observed among our participants is also higher than those reported among adults with unspecified HIV status in Nigeria and the Gambia, where 26 % of adults >18 years of age and 21 % of mothers of 12-month-old infants were colonized, respectively [5, 30]. These variations may be partially explained by the broth enrichment step used in this study, which was not done in other studies from Africa. We used broth enrichment before plating because it has been shown to increase recovery of pneumococci from respiratory specimens [23]. In addition, all participants in our study were parents of young children who live together in the same compound, which may result in frequent transmission of pneumococci. Other unspecified differences in the populations studied in other African pneumococcal colonization studies (e.g. recent illness or tobacco smoke exposure) may also have contributed to the differences observed in colonization rates. The high colonization rate among our participants with unknown HIV status (35.8 %) suggests that undocumented HIV-infection may have been common among adults in this group. Two groups known to be at higher risk of HIV are those who refused HIV testing and those who were recent in-migrants to the study area. [31]

In this study HIV-infected parents were over two times more likely to be colonized with pneumococci than HIV-uninfected parents living in compounds with an HIV-infected parent, when adjusting for other risk factors. Because the mechanism of mucosal protection against pneumococcal colonization is T-cell dependent, HIV-infected persons with low CD4 counts may be at highest risk for colonization and consequently for invasive disease. CD4 counts less than 350 have been associated with increased risk of invasive disease in some studies [6]; however, the link to colonization is less well established [28, 29, 32]. No differences in colonization prevalence by CD4 count were previously found among HIV-infected adults in studies conducted in Kenya, Brazil, and South Africa [25, 29, 33]. We found HIV-infected parents with counts ≥250 to be somewhat protected from pneumococcal colonization. Although our data were limited, we did not see a protective effect with usage of HAART or attendance at an HIV clinic among HIV-infected parents, which has been described before [33]. Besides HIV infection, we also observed a significant association between pneumococcal colonization and cooking location where persons who described cooking in an area separate from their sleeping area were less likely to be colonized. Exposure to indoor air pollution has been linked to adverse health outcomes including pneumonia in the developing world [34] however the relationship with pneumococcal colonization is less well established [35, 36]. We did not observe an association between colonization and other established risk factors for pneumococcal carriage including age, tobacco smoke exposure, or the number of children <5 years in the home [37, 38].

We detected 41 different serotypes in this rural Kenyan population, with a greater degree of diversity observed among HIV-uninfected compared to HIV-infected participants. This finding is consistent with the greater diversity of invasive serotypes observed among HIV-uninfected compared to HIV-infected adults in South Africa [12] and will have implications for understanding changes in pneumococcal ecology and serotype replacement after vaccine introduction [39]. The most frequent colonizing serotypes observed among HIV-infected parents in our study (3, 16 F, 19 F, 23 F) are similar to findings from other surveys conducted among HIV-infected adults in Nigeria, South Africa, and Uganda [26–28] and among HIV-infected children in Kenya and Tanzania [35, 36]. HIV-infected parents were slightly more frequently colonized with PCV10-type and PCV13-type pneumococci than HIV-uninfected parents, although this difference was not statistically significant. The baseline colonization rate among these groups will be important to understanding the impact of vaccine introduction in Kenya and will complement data on invasive disease as it becomes available. The effect of PCV10 introduction on the prevalence of serotypes 3 and 19A will be particularly important. In our study serotype 3 was the most frequently carried serotype, although it is not covered by PCV10. Serotype 19A, also not included in PCV10, was not detected in our study but increased in some countries after PCV7 introduction [40–43]. Still, as early data documenting the impact of PCV10 on invasive disease among Kenya children are becoming available [44], the potential for indirect impact of the vaccine on pneumococcal colonization and disease among non-vaccinated groups appears promising. In the U.S., HIV-infected persons experienced a 91 % drop in vaccine-type IPD after vaccine introduction [7].

Our study had several potential limitations. Conducting the study over an 8-week period might only provide a snapshot of colonization in this population, as pneumococcal disease is transient and has been shown to vary by season in some studies [35, 45] though not others [46]. Second, much of our HIV-related data pre-dated enrollment in the carriage study by over 1 year which may have resulted in misclassification, as HIV-uninfected may have become HIV-infected during the time since their negative test. In the Asembo area, the HIV incidence is estimated to be approximately 1.2 % per year [personal communication, KEMRI/CDC]. Similarly, data on CD4 counts may have been inaccurate as these are also time-sensitive and can change over the course of HIV infection and with use of HAART.

## Conclusions

Characterizing the direct effect of PCV among children targeted to receive vaccine, and assessing the indirect vaccination effect among high-risk groups in Kenya will have important policy implications in Africa. Because HIV-infected persons have an impaired immune response to polysaccharide vaccine, and conjugate vaccines are not yet approved for their use in adults in most countries [47, 48], the indirect effects of PCV introduction in Kenya will be critical in preventing disease in this group.

Sustaining long term funding for vaccination programs in early-adopting countries like Kenya and encouraging other countries to adopt PCV will require demonstration of vaccine impact. More potential factors may lead to a different herd effect with introduction of PCV in Africa than in high-income countries, including a potentially lower rate of vaccine coverage, different socio-economic conditions, and a variety of additional factors which could influence the immunologic responses or protective effect among vaccinated children (e.g. younger target age-group, and higher prevalence of malaria and malnutrition) [1, 49]. The baseline data presented in this report will be compared with ongoing analyses of pneumococcal carriage rates in HIV infected parents over three years following introduction of PCV10 in infants in Kenya. Once this investigation is completed, the findings will contribute to a growing body of data on PCV impact that will be critical for decision-making regarding sustained pneumococcal immunization in Africa.

## Abbreviations

CDC: Centers for Disease Control and Prevention
HAART: Highly Active Antiretroviral therapy
HDSS: Health and Demographic Surveillance System
HIV: Human Immunodeficiency Virus
IPD: Invasive Pneumococcal Disease
KEMRI: Kenya Medical Research Institute
NP: Nasopharyngeal
OP: Oropharyngeal
PBIDS: Population Based Infectious Diseases Surveillance
PCV: Pneumococcal Conjugate Vaccine
PCV10: 10-valent Pneumococcal Conjugate Vaccine
PCV13: 13-valent Pneumococcal Conjugate Vaccine
STGG: Skim milk-tryptone-glucose-glycerol
SDI: Serotype Diversity Index
